# The systemic impact of acute exacerbations of COPD requiring hospitalisation: a narrative review

**DOI:** 10.1183/23120541.01725-2025

**Published:** 2026-06-08

**Authors:** George Mills, Joseph Kibbler, Lorna Latimer, Hamish J.C. McAuley, John Steer, Neil J. Greening

**Affiliations:** 1 Centre of Exercise and Rehabilitation Science, NIHR Leicester Biomedical Research Centre – Respiratory and Infection Theme, Leicester, UK; 2 Department of Respiratory Sciences, University of Leicester, Leicester, UK; 3 Translational and Clinical Research Institute, Newcastle University, Newcastle upon Tyne, UK; 4 Department of Respiratory Medicine, Northumbria Healthcare NHS Foundation Trust, North Tyneside General Hospital, North Shields, UK

## Abstract

Acute exacerbations of COPD (AECOPD) are a major cause of morbidity and mortality, with systemic effects beyond the lung complicating treatment and recovery. Understanding the multisystem impact of AECOPD, as well as established and emerging approaches to mitigate these issues, is important for optimising patient management. This review explores the systemic impact of hospitalisation with AECOPD, such as the primary pulmonary and systemic inflammatory burden, the effects of prolonged inactivity and consequences of specific treatments. Complications include an increased risk of cardiovascular events, hyperglycaemia, cognitive impairment and confusion, and loss of muscle mass and function. These effects share several key pathophysiological features, including high-grade systemic inflammation and hypoxaemia-induced impairments to cellular energy production. Many of these systemic consequences have substantial implications for patients’ health and quality of life beyond that of the primary lung insult. As such, recognising and treating these effects are crucial in the management of AECOPD. These treatments should be specified based on the presenting features, though cornerstones include regulation of blood oxygen and glucose levels, limited use of glucocorticoids, nutritional management and post-exacerbation pulmonary rehabilitation. Several emerging pharmacological compounds have demonstrated promise in addressing some systemic effects. The systemic impact of AECOPD is widespread, with major consequences for short- and long-term outcomes. By mapping this diverse body of evidence, this narrative review highlights the need to recognise and treat systemic effects alongside the primary pulmonary insult and identifies promising avenues for novel management strategies.

## Shareable abstract

**This narrative review examines the systemic consequences of hospitalisation with an acute exacerbation of COPD – highlighting underlying mechanisms, clinical relevance and emerging treatment strategies** https://bit.ly/4rdOoKo

## Introduction

COPD affects over 400 million individuals globally and ranks as the third leading cause of death worldwide [1]. For many individuals living with COPD, acute exacerbations (AECOPD) or “flare ups” are a frequent occurrence. Cases of AECOPD requiring hospital admission carry an immediate mortality rate of 6.7% in Western countries [2], with the risk of longer-term complications and impairments amongst survivors representing a major concern.

Exacerbations cluster in time and are temporally associated with other acute illnesses, contributing to high hospital readmission rates [3]; ∼25% of patients are readmitted within 30 days and nearly 50% within 90 days [4]. Beyond the primary insult to the lungs, the combined effect of reduced physical activity, physiological stress and the impact of certain treatments (*e.g.* oral corticosteroids) increases the potential for systemic consequences [5, 6]. Understanding how these episodes impact bodily systems, along with their influence on one another, is essential for effective management. This narrative review aims to summarise the latest evidence regarding the clinical manifestations, underlying pathophysiology and management of the systemic consequences of AECOPD, with a focus on severe exacerbations requiring hospitalisation.

## Literature search

A structured, narrative approach was adopted in accordance with the SANRA framework [7]. Searches were conducted in October 2024 and updated in January 2026, with no minimum date restriction, on PubMed (MEDLINE) and Embase. Published clinical guidelines were identified directly *via* Google and reference lists of journal articles. No limits were set on study design and articles in any language were considered. Previous literature reviews were included and their reference lists searched for relevant studies.

All searches began with a core set of terms: “Chronic Obstructive Pulmonary Disease/” (MeSH), “COPD.mp.”, “(chronic obstructive adj3 (lung disease or pulmonary disease)).mp.”, “emphysema.mp.”, “bronchitis.mp.” and “(chronic bronchitis or chronic airflow limitation).mp.” to identify COPD-related studies. This was combined with the terms “Exacerbation/” (MeSH), “(acute adj3 exacerbation*).mp.” and “(worsen* or flare-up* or acute deterioration).mp.” to identify studies in AECOPD populations. Search terms indicating hospitalisation were not employed to avoid excluding studies in hospitalised cohorts that were not indexed as such. From this, additional search terms relevant to each individual topic of this review were incorporated and are available in the online supplementary material. Titles, abstracts and full texts were screened for eligibility by authors for each section.

From an initial 3424 total records identified, 601 duplicates and 32 study protocols were removed, leaving 2806 titles and abstracts for screening. At this stage, studies focusing on stable COPD, nonacute, non-COPD populations, or reporting only respiratory (nonsystemic) outcomes were excluded, leaving the studies reported in this review. A further six studies and one guideline document were identified and included following the updated literature search. As the aim of this review was to map the breadth of available evidence, where multiple studies addressed the same outcome, findings were synthesised narratively to emphasise consistent trends rather than to exhaustively summarise all available data.

## Evidence review

**FIGURE 1:**
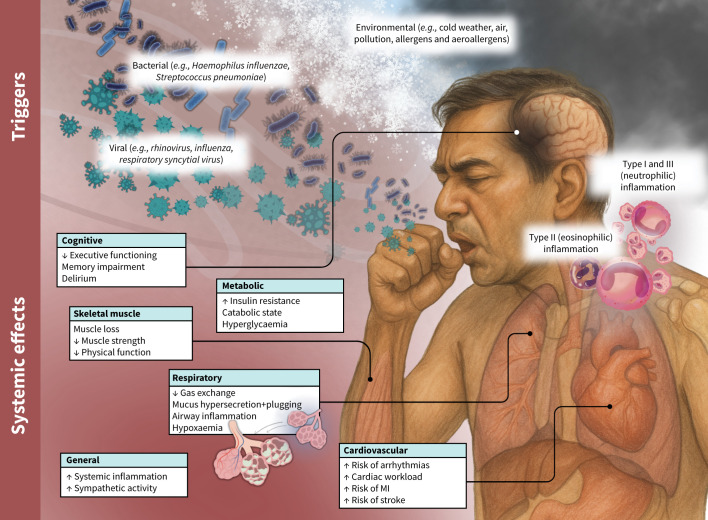
The systemic effects of acute exacerbations of COPD. Effects are seen across multiple organs, including heart, skeletal muscle, brain and airways. MI: myocardial infarction. Illustration of patient generated with assistance of AI (OpenAI, 2025); figure adapted and composed by the authors*.*

Severe exacerbations of COPD are driven by a cascade of physiological responses that extend far beyond the airways. In response to an initial exposure or trigger, a local inflammatory and immunological reaction ensues, manifesting primarily as increased bronchoconstriction and mucus hypersecretion (figure 1) [8]. For the patient, this results in increased airway resistance and greater work of breathing, contributing to impaired gas exchange within an already compromised system.

In the acute phase, low oxygen supply leads to a shift from aerobic towards anaerobic glycolysis, restricting adenosine triphosphate (ATP) production for high-energy–demanding organs, while hypoxia-induced signalling pathways such as HIF-1α and nuclear factor-κB trigger microvascular dysfunction, increasing permeability and leading to tissue oedema [9]. The diversion of blood flow from other organs to the thoracic muscles, known as “respiratory steal” syndrome, [10] further compromises perfusion and can have pronounced effects in COPD patients where poor capillarisation and chronic vascular insufficiency are more common. Meanwhile, carbon dioxide retention (hypercapnia) in the blood causes respiratory acidosis and impairs cellular enzyme activity [11]. Together, these changes can precipitate multi-organ failure [12].

Importantly, these physiological stressors occur in a population with already established and often advanced disease, which is usually associated with multiple long-term conditions: referred to as multi-morbidity. The resultant increased vulnerability of these systems means that a sudden influx of inflammatory mediators may have more profound consequences on the organs. When the local inflammatory response in the airways is substantial enough, the “spill over” into systemic circulation marks the beginning of many of the effects explored in this review [13, 14]. The response of individual organs impact one another, and the influence of existing comorbidities means that this response is unpredictable and specific to the individual.

## Cardiovascular complications

### Adverse cardiovascular events and AECOPD

A remarkable 28 out of every 100 hospitalisations with AECOPD are followed by an adverse cardiovascular (CV) event [7]. The importance of an exacerbation in the occurrence of adverse cardiovascular events is underlined by the EXACOS-CV programme: in the Italian cohort, the adjusted hazard ratio (aHR) for acute coronary syndrome during days 1 to 7 following an AECOPD was 9.83 (95% CI 8.72–11.07), decreasing to 1.61 (1.21–2.13) by days 8 to 14 [15]. Heightened cardiovascular risk does not return to baseline following COPD exacerbations and remained elevated for up to 365 days [16]. Similar risk patterns were observed in the UK EXACOS-CV cohort, with aHRs of 8.31 (6.79–10.2) for heart failure (HF) and 12.2 (10.1–14.8) for arrhythmia in the 2 weeks following hospitalisation [17]. Risk was also elevated post-moderate exacerbation, but to a lesser degree, implying greater CV risk with increasing AECOPD severity.

The acute cardiovascular risks of AECOPD occur against a background of substantial unrecognised chronic disease. For example, while just 1.7% of patients in the UK EXACOS-CV cohort had diagnosed HF at baseline, 11.3% experienced a HF event during follow-up. As anticipated, when COPD patients with no known cardiovascular disease (CVD) are investigated systematically, diagnostic pick-up is considerable: ∼15% have significant undiagnosed left ventricular systolic dysfunction [18] and between 28% and 60% of patients have unrecognised obstructive coronary artery disease [19, 20]. Disappointingly, even when comorbid CVD *is* diagnosed, it is often undertreated: patients with COPD are significantly less likely to receive β-blocker, aspirin or antiplatelet treatment post-myocardial infarction [21].

### Pathophysiology

A range of physiological changes occur during AECOPD that adversely affect the CV system. Worsening hypoxaemia reduces myocardial oxygen supply at a time when demand is increased due to concurrent tachycardia, systemic inflammation and respiratory effort. This leads to myocardial ischaemia, as measured by cardiac troponin elevation, in approximately half of hospitalised patients [22]. Hypoxaemia also activates the coagulation cascade and induces neutrophils to hyper-secrete histotoxic proteins, causing vascular injury and predisposing to acute thrombotic events [23].

The increase in systemic inflammation seen during AECOPD heightens cardiovascular risk further. Exacerbations are associated with elevated circulating interleukin-6 (IL-6), C-reactive protein, leukocytes and neutrophils. These pro-inflammatory markers correlate with increases in arterial stiffness and troponin release in AECOPD, implicating inflammation as a mediator of acute myocardial injury [24].

Furthermore, dynamic hyperinflation during AECOPD causes reduced diastolic cardiac filling *via* increased intrathoracic pressure, significantly reducing left ventricular preload, [25] and contributing to the acute cardiac dysfunction evidenced by increased N-terminal pro-brain natriuretic peptide (NT-proBNP) levels and clinically relevant fluid overload [26]. Additionally, dynamic increases in pulmonary pressures, likely to be mediated by pulmonary vasoconstriction during AECOPD events, correlate with rehospitalisation and death, highlighting the importance of acute cardiodynamic changes [27]. Finally, sympathetic nervous system activity increases during AECOPD, potentiated by the increased use of bronchodilating drugs such as salbutamol [28, 29]. Increased sympathetic activity drives arrhythmogenicity and myocardial oxygen demand and is associated with an increased risk of hospitalisation and mortality in COPD [30].

Thus, critical factors that link the presence of COPD to the presence of heart disease and acute cardiodynamic changes – hypoxaemia, inflammation, lung hyperinflation and autonomic derangement – have been demonstrated to be intensified during the exacerbation period, with a correspondingly increased temporal risk for adverse cardiac events, rehospitalisation and death [27, 31].

### Management

Recent randomised and meta-analysis data shows that, in selected patients, triple inhaled therapy (inhaled steroid, long-acting β-agonist, long-acting muscarinic antagonist) reduces exacerbation frequency in COPD [32, 33]. Secondary analyses have suggested possible reductions in all-cause mortality, but these studies were not powered to detect cardiovascular-specific outcomes. By reducing exacerbations, triple therapy may plausibly contribute to lowering downstream cardiovascular risk [34].

Observational data showed that certain cardiovascular therapies (β-blockers and statins) were associated with lower mortality risk in COPD. However, in randomised controlled trials, when patients with an indication for these therapies were excluded, no effect on mortality or exacerbation rate was seen [35–37]. This highlights that these trials were not designed or powered to evaluate cardiovascular outcomes, and suggests that the benefit seen in observational trials may be due to the presence of underdiagnosed CVD in COPD.

This speaks to the more fundamental problem of a reliance on symptom-driven diagnostics for CV comorbidity. This existing approach to addressing CVD in COPD is inadequate: 71% of COPD patients with severe coronary artery stenosis have no chest pain, and severe left ventricular systolic dysfunction during AECOPD is undetectable by clinical assessment in 67% [20, 38]. As a result, many patients with treatable CV pathology are missed during both acute care and outpatient follow-up [18].

Given the window of elevated cardiovascular risk after AECOPD, and the unacceptably high rates of undiagnosed CVD in COPD, clinicians need to make every contact count and proactively investigate for CVD. Additionally, in patients with known CVD, ensuring that all guideline recommended therapies are being used will improve cardiovascular outcomes. Priority tests and interventions to achieve this are summarised in table 1. Encouragingly, the 2026 Global Initiative for Chronic Obstructive Lung Disease (GOLD) guidelines reflect a shift in attitude by stating that measurement of NT-proBNP and troponin may be advisable during AECOPD [39]. In the future, wider awareness of the emerging concept of “cardiopulmonary risk” will increase integration of care between respiratory and cardiology services, reduce diagnostic and treatment gaps, and improve patient outcomes [40].

**TABLE 1:** Priorities and strategies at each encounter to improve cardiovascular (CV) outcomes post-acute exacerbation of COPD (AECOPD)

Patient–clinician encounter	CV risk mitigation priorities	Investigations/interventions required
**Primary care COPD review**	- Assess CV comorbidity accurately - Treat known CVD optimally - Smoking cessation^#^	- Optimise CV medication - Lipids, QRISK score^¶^±statin - BP, pulse check±ECG - Diabetes screening
**Admission with AECOPD**	- Identify and treat concurrent decompensated HF/ACS/arrhythmia	- ECG ±troponin - NT-proBNP±echocardiogram - Urgently assess and optimise management of new CVD
**Respiratory outpatient clinic**	- Consider undiagnosed CVD in deteriorating patients	- NT-proBNP±echocardiogram CTCA if suspected coronary artery disease - Referral to cardiology if new heart disease diagnosis or ongoing clinical concerns

CVD: cardiovascular disease; BP: blood pressure; ECG: electrocardiogram; HF: heart failure; ACS: acute coronary syndrome; NT-proBNP: N-terminal pro-brain natriuretic peptide; CTCA: computed tomography coronary angiogram. ^#^: smoking interventions should take place at every interaction. ^¶^: while QRISK score remains a standard tool for primary care CV risk assessment, recent evidence indicates that QRISK3 may underestimate CV event risk in patients with COPD [104]. ECG-derived indices such as the Cardiac Infarction Injury Score (CIIS) and P pulmonale have been proposed as additional markers of CV risk [105].

## Cognitive impact

### Cognitive impairment and delirium in AECOPD

Cognitive impairment, defined as measurable deficits in memory, attention or executive function, is common during AECOPD and is often transient in nature [41, 42]. Within 24 h of admission, cognitive impairment – defined as a score of <26 in the Montreal Cognitive Assessment (MoCA) – was identified in 76% (mean±SD score 22.2±3.9) of individuals admitted with AECOPD to a respiratory ward in New Zealand [42]. This prevalence is notably greater than that of stable COPD populations without hypoxaemia, at ∼36% (mean±SD score 25.6±2.9) [43]. Higher rates, exceeding 90%, have been reported during early AECOPD hospitalisation in similar age groups [44, 45].

Beyond this period, conflicting evidence exists as to the trajectory of cognitive function among patients. López-Torres and colleagues [41] found significant improvements in MoCA score during a median 9-day admission (19.28±2.08 to 23.91±3.74), while Poot and colleagues [42] observed no change between <24 h, 48–72 h and 6–8 weeks post-discharge. Others similarly reported stable MoCA performance up to 3 months following AECOPD discharge [46, 47].

Tools such as the Confusion Assessment Method (CAM) are sensitive to detect more acute and fluctuating conditions such as delirium, which have been reported in 15% of individuals admitted with AECOPD to a hospital in the United Kingdom [44]. Though less common, delirium represents a serious complication in severe AECOPD and serves as a strong predictor of 1-year mortality [48], with systemic corticosteroid treatment potentially increasing the risk in susceptible patients [49].

### Pathophysiology

Markers of COPD and AECOPD severity, such as forced expiratory volume in 1 s, arterio-alveolar gradient and partial pressure of carbon dioxide have shown weak correlations with neuropsychological test performance in previous work [46]. Interestingly, indicators of cerebrovascular health quantified by the Framingham Stroke Risk Score have similarly failed to provide primary explanations for cognitive impairment in AECOPD [46]. As a result, the exact mechanisms that may trigger or exacerbate mild cognitive impairment during hospitalisation with AECOPD remain unclear.

Hypoxaemia in COPD is associated with decreased attention, slower mental speed and compromised executive functioning [50]. Whilst underlying mechanisms have been derived mostly from animal models [51], there is a firm understanding of oxygen dynamics during an acute exacerbation – in that reduced gas exchange coupled with an increase in tissue oxygen demand means that global hypoxia is prevalent [52]. The switch to anaerobic glycolysis in the brain triggered by hypoxic conditions can cause decreased ATP production which could result in tissue acidosis and subsequent neuronal damage [51]. Intracellular ATP and ADP may also be reduced because of an increase in adenosine concentrations [53], leading to impaired synaptic transmission. In addition to hypoxia, it has been hypothesised that the systemic build-up of pro-inflammatory mediators during AECOPD may play a role, disrupting the integrity and structure of the blood–brain barrier and causing insult to the brain [54]. Direct alterations to the microvasculature of the brain have also been implicated, though little human evidence is available in this area to date [46].

### Management

Despite observational findings of hypoxaemia and mild cognitive impairment in AECOPD, acute oxygen supplementation (<24 h) has failed to reverse deficits in neuropsychological test performance [55]. Longer term (>6 months) oxygen therapy in hypoxic COPD has demonstrated minor efficacy, but the impact on health-related quality of life may be minimal [56]. Other proposed treatments in stable COPD have included cognitive training, though this is likely to be too demanding to be implemented during an exacerbation and a previous randomised controlled trial found no improvements in cognitive performance over 6 months [57].

Following a 6-week post-exacerbation pulmonary rehabilitation programme, France and colleagues [47] reported an increase in mean MoCA score by 1.6±2.4 points. Despite no present minimal clinically important difference (MCID) established in COPD, this exceeds the MCID for the MoCA established for stroke rehabilitation [58]. Given the broader benefits of exercise and physical activity and its effectiveness in the management of mood disorders [59], exercise-based rehabilitation should be considered the first-line treatment for mild cognitive impairment following AECOPD in the absence of underlying pathology.

It is important to note that cognitive impairment and delirium may have wider implications for other aspects of care. Patients may have difficulty understanding treatment advice, such as medication adherence, inhaler technique or engagement with follow-on services such as post-exacerbation pulmonary rehabilitation. Overall, current guidelines provide little specific advice on cognitive impairment or delirium during AECOPD. Evidence–based delirium practices including systematic risk assessment, regular screening using validated tools and nonpharmacological multicomponent interventions (orientation, hydration, infection control, pain management, mobility) should be employed [60, 61].

## Skeletal muscle

### Changes in muscle mass, strength and function

Established deficiencies in muscle mass and strength have been noted in ∼22% (95% CI 15–31%) of individuals with COPD [62]. Quantifying the impact of acute exacerbations has proved challenging, complicated by the severity of the acute illness and the absence of pre-hospitalisation measures. Consequently, there has been a lack of observed changes in muscle strength between admission and discharge when assessed *via* dynamometry [63–65] and the 5-repetition sit-to-stand test [66]. Indeed, much of this evidence is derived from control arms of interventional trials and are therefore likely underpowered to detect meaningful change [63, 65–67].

Studies that have prospectively assessed these variables provide an alternative perspective. Amongst 52 patients admitted to a respiratory care unit with AECOPD across two hospitals in Granada, Spain, maximal isometric quadriceps strength decreased from 9.45±3.29 kg to 7.40±3.11 kg over a mean of 12 days admission [68]. Further, at 1 month post-admission, a continued decline in muscle strength was observed to 6.17±3.54 kg. Changes in quadriceps muscle size were assessed by McAuley and colleagues *via* ultrasound in 55 individuals between admission (within 48 h) and discharge (median day 5) [69]. Over the study period, quadriceps thickness reduced by 8.3% (−2.28 mm, 95% CI −1.16– −3.40 mm). Importantly, when followed-up at 6–8 weeks, quadriceps thickness was still markedly reduced compared to admission (−8.5%; −2.52 mm, 95% CI −1.09– −3.97 mm). These studies provide evidence of the acute impact of AECOPD hospitalisation on skeletal muscle tissue.

### Pathophysiology

**FIGURE 2:**
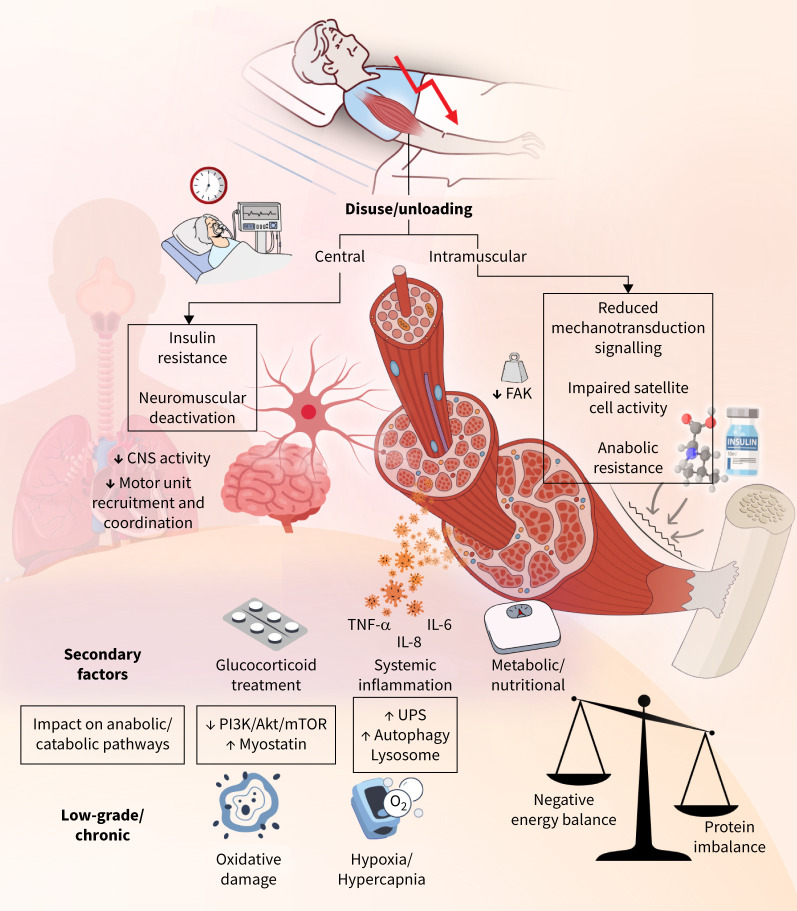
Mechanisms of skeletal muscle dysfunction during hospitalisation with an acute exacerbation of COPD. PI3K/Akt/mTOR: phosphoinositide 3-kinase/protein kinase B/mechanistic target of rapamycin; TNF-α: tumour necrosis factor-α; IL-6: interleukin-6; IL-1β: interleukin-1β; FAK: focal adhesion kinase; CNS: central nervous system; UPS: ubiquitin–proteasome system.

Physical inactivity and the unloading of ambulatory muscles likely plays a central role in muscle dysfunction during AECOPD; weight-bearing movement accounts for a median of 7% to 9% of daily activity during admission and is positively correlated with quadriceps force production [70]. Disuse models in healthy humans – most commonly bed rest or single limb immobilisation – provide insight into the mechanisms driving this immobilisation-induced muscle atrophy. Of note, decreased activation of the Phosphoinositide 3-kinase/Protein kinase B/Mechanistic target of rapamycin (PI3K/Akt/mTOR) pathway due to increased insulin resistance and impaired Insulin-like Growth Factor 1 (IGF-1) signalling may contribute to the depression of muscle protein synthesis (MPS) seen during unloading (figure 2) [71]. However, it is unclear how much direct influence this pathway has on MPS dynamics in humans [71]. Evidence of the reduced activation of mechanosensitive proteins embedded within muscle fibres, such as focal adhesion kinase (FAK) [72], may partially explain the differential loss of mass seen in primary muscles involved in postural support and lower-limb movement [73].

During AECOPD, elevated levels of pro-inflammatory cytokines such as tumour necrosis factor-α, interleukin-6 (IL-6) and IL-1β alongside the presence of increased oxidative stress may contribute further to muscle loss by increasing protein degradation. Similarly, although less well understood mechanistically, chronic hypoxia is associated with reduced muscle mass, potentially mediated *via* pro-inflammatory pathways [74]. While effective for reducing inflammation, treatment with glucocorticoids has well established suppressive effects on muscle anabolism and triggers protein degradation, primarily *via* the ubiquitin proteasome system [75]. Despite this evidence, the relative contribution of altered rates of MPS and breakdown on muscle mass loss during AECOPD is not well understood.

### Management

Despite being the most established method of stimulating muscle fibre hypertrophy in healthy individuals and stable COPD, resistance training has demonstrated mixed efficacy during AECOPD [65, 67, 76, 77]. This is most likely explained by the severity of the acute condition and limited time window for intervention, as well as inability of patients to achieve muscle overload.

In order to overcome these limitations, pharmacological methods of preventing muscle atrophy in AECOPD have been explored. Ferreira and colleagues [78] administered intramuscular testosterone and oral stanozolol for 6 months in stable individuals, who gained a mean of 1.8±0.5 kg body weight with an increase in lean mass, whereas the control group lost −0.4±0.2 kg. Several studies since have explored similar regimes, with a recent meta-analysis reporting a standardised mean difference of +0.98 (95% CI 0.24–1.72) kg in lean body mass with testosterone use in COPD [79]. However, significant study heterogeneity and a lack of inpatient data prevent conclusions on safety and efficacy. Alternative therapies include neuromuscular electrical stimulation (NMES), which delivers superficial electrical currents to skeletal muscle to induce contraction, providing a nonvolitional alternative to resistance training and carrying a low ventilatory and metabolic load [80]. A 2024 meta-analysis found a potential positive effect of NMES on limb muscle strength during AECOPD, though interpretation was limited due to a high risk of bias [81].

**FIGURE 3:**
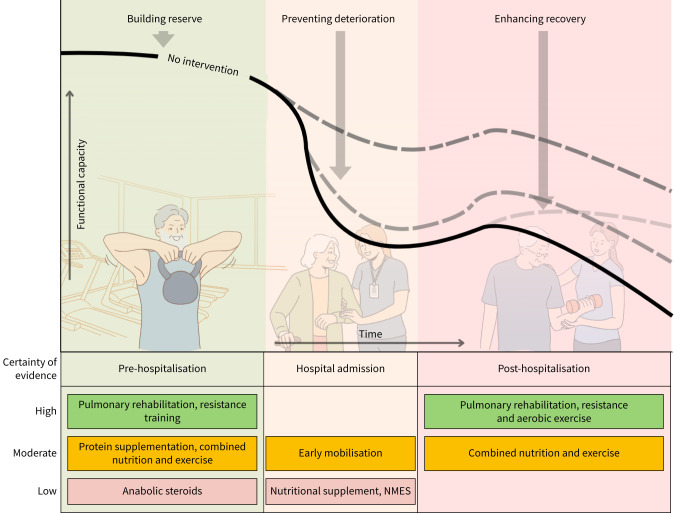
Interventions to enhance functional capacity before, during and after AECOPD and the certainty of their evidence. NMES: neuromuscular electrical stimulation.

Beyond this, the development of new therapies aimed at improving muscle integrity and building physical reserve before an AECOPD event presents a promising opportunity (figure 3), though to date, studies have failed to detect concomitant improvements in functional performance [82, 83]. Novel agents targeting the neuromuscular junction have been highlighted as a future area of interest for treating muscle dysfunction in the general population, with neuromuscular efficiency emerging as a potential therapeutic target [84]. However, there is currently a lack of substantive evidence for treatments targeting acute muscle dysfunction in AECOPD.

Though effective in the nonacute state, professional bodies consistently recommend against initiating exercise-based rehabilitation during the inpatient period [85, 86]. Post-exacerbation pulmonary rehabilitation, initiated within 4 weeks of discharge, remains the most evidence-based approach [39, 85, 86].

## Metabolic impact

### Effects of AECOPD on metabolism

During AECOPD, the elevation of proinflammatory cytokines causes significant disruptions to metabolic homeostasis [87]. Observations have shown that ∼50% of individuals meet the threshold for hyperglycaemia within 24 h of hospital admission [88, 89]. This is important, as there are positive associations between hyperglycaemia and risk of death, length of hospital stay and failure of noninvasive ventilation treatment in AECOPD [88, 89]. Although many of these observational studies are subject to limitations, some findings suggest that this relationship may be independent of corticosteroid use prior to admission, or the presence of diabetes mellitus [89].

From the initial onset of symptoms prior to AECOPD hospitalisation, caloric intake may be reduced by 28% from habitual levels and is further diminished by the day of admission [90]. Moreover, an increase in metabolic rate seen in some individuals, driven primarily by the severe systemic inflammatory response and subsequent impact on thermogenesis, may contribute to an overall negative energy balance [91]. This imbalance has well-documented downstream effects on skeletal muscle tissue, with adipose and muscle tissue loss occurring at a ratio of 4:1 under moderate hypocaloric conditions in healthy older people [92]. Whilst this reflects normal metabolic adaptation, the presence of a substantial negative energy balance will result in an acceleration of skeletal muscle tissue loss, further impairing metabolic health and recovery. It should be noted that under extreme hypocaloric conditions, individuals with very low body fat carry a particularly high risk of muscle loss, as the body begins to break down muscle rapidly to meet its basic energy demands.

### Pathophysiology

During the acute phase, elevated levels of proinflammatory cytokines trigger an increase in hepatic glucose production which is further exacerbated by an increase in circulating catecholamines [93]. Upon admission to hospital, treatment with glucocorticoids such as prednisolone is usual practice, a recognised side-effect of which is increased endogenous glucose production [94]. Together, this may contribute to the development of peripheral insulin resistance. Newer treatments such as SGLT2 inhibitors are now seen more widely in patients with diabetes mellitus, which is commonly associated with COPD. Current recommendations are that these medications are stopped during acute illness when admitted to hospital, potentially inducing further hyperglycaemia [95].

Hormonal dysregulation can also lead to impairments in metabolic processes in AECOPD. During an exacerbation, concentrations of leptin – a hormone central to the regulation of energy balance – are elevated, driven mostly by systemic inflammation and higher doses of glucocorticosteroids [96]. Disruptions in leptin signalling have been implicated in the alterations in energy balance seen during a severe AECOPD [96], contributing to the loss of skeletal muscle tissue.

Further to hormonal dysregulation, worsening breathlessness is commonly experienced by patients during AECOPD and may be further exacerbated by the sensation of fullness after meals. In patients with significant hyperinflation, gastric distension following the consumption of food may theoretically further impair diaphragmatic excursion by splinting the diaphragm, increasing the work of breathing and contributing to early satiety. However, the extent to which this phenomenon contributes to reduced energy intake is inconclusive [97, 98].

### Management

There are currently no published guidelines on the measurement of blood glucose during AECOPD, though recommendations have included screening on admission and at regular intervals (three to four pre-prandial assessments per day) during corticosteroid treatment [99]. In terms of active approaches, optimisation of oral intake during the early stages of hospitalisation when protein and total caloric intake is likely to be low, and avoiding unnecessary corticosteroid exposure beyond recommended short courses, should be encouraged. Nutritional supplementation during AECOPD is feasible, but lacks efficacy in terms of preserving muscle strength, and has mixed results for lung function improvement [63].

Regulation of glucose levels using insulin has not been specifically tested in patients with COPD. However, a number of guidelines for hyperglycaemia in both critical care and noncritical settings recommend non-tight control of blood glucose with instigation of treatment above 180 mg·dL^−1^ (>10.0 mmol·L^−1^) but with careful monitoring to avoid hypoglycaemia [100]. Furthermore, optimisation of glycaemic control among patients with only moderately raised blood sugar may be achieved *via* basic mobilisation alongside reduced carbohydrate intake. Current guidelines for the specific management of hyperglycaemia in AECOPD are lacking, though many recognise the elevated risk during AECOPD and emphasise short courses of oral corticosteroids to reduce the risk of adverse metabolic effects [85]. For energy imbalance, The British Association for Parenteral and Enteral Nutrition (BAPEN) offer broad guidance on nutritional intervention during AECOPD, with the aim being to minimise loss of weight and muscle mass by increasing protein and energy intake and using oral nutritional supplements where necessary [101].

## Clinical implications and future directions

The potential for disruption across multiple systems during AECOPD highlights the importance of effectively targeting nonpulmonary factors. The high rates of adverse cardiac events, which spike shortly after an AECOPD, and the concerning rates of underdiagnosis of cardiac disease, mean that clinicians caring for AECOPD should proactively investigate for all treatable factors that increase cardiopulmonary risk. Moreover, specialty-based models of care may be inefficient and time-restricted during COPD exacerbations – patients with multimorbidity may deteriorate or be readmitted before other specialists can engage. A nationwide registry study in Denmark reported that 60% of subsequent readmissions after an AECOPD event were attributable to nonrespiratory causes, and the likelihood of readmission rose by nearly 50% with each additional comorbidity [102]. This underscores the need for respiratory clinicians to adopt a multi-organ focus in the recognition and management of AECOPD.

Features such as muscle atrophy and reduced nutrient intake may be challenging to actively treat during AECOPD. Future directions should therefore consider pharmacological strategies to mitigate these effects, particularly as an adjunct to established nonpharmacological treatments where suitable (table 2). Beyond the acute phase, a substantial body of evidence has confirmed the effectiveness of pulmonary rehabilitation for improving physical function and health-related quality of life, which should be commenced within 1 month following discharge [103]. Ongoing management of comorbidities, such as metabolic and CVDs, should form part of a comprehensive strategy to reduce the risk of adverse outcomes from future exacerbations.

**TABLE 2:** Overview of treatments for the systemic effects of acute exacerbation of COPD (AECOPD)

Systemic consequence	First line/established treatments	Emerging interventions/lower certainty of evidence
**Cardiovascular** **(*e.g.* ACS, arrhythmia, HF)**	Controlled oxygen therapy (*S*_pO_2__ between 88% and 92%) to reduce myocardial oxygen supply–demand mismatch Identification and management of CV risk factors: smoking cessation, QRISK scoring, lipid profile, ECG/pulse check, BP control Optimisation of established CVD: guideline-directed therapy for HF, CAD and AF (β-blockers, antiplatelets, ACEi/ARNI, MRA, SGLT2i, anticoagulation as indicated) Use of inhaled triple therapy in patients with high exacerbation risk and evidence of Type II inflammation (to reduce exacerbation burden, rather than direct CV event prevention)	NT-proBNP and troponin measurement during AECOPD to aid detection of occult cardiac dysfunction Proactive case finding with advanced diagnostics (echocardiography, cardiac imaging) Multidisciplinary cardiopulmonary risk screening pathways
**Cognitive** **(*e.g.* delirium, executive dysfunction)**	Delirium screening using validated tools (*e.g.* CAM) Controlled oxygen therapy in presence of hypoxaemia Noninvasive ventilation (as per guidelines) in hypercapnic, acidotic AECOPD to correct CO₂-related encephalopathy Effective treatment of initial infection/underlying cause (monitoring of infection markers, hypoxaemia, hypercapnia and electrolytes) Pulmonary rehabilitation (<1 month post-exacerbation) to support cognitive recovery	Use of structured cognitive assessments (*e.g.* MoCA, Mini-ACE) post-AECOPD
**Musculoskeletal** **(*e.g.* acute sarcopenia, reduced function)**	Pulmonary rehabilitation (<1 month post-exacerbation) Resistance training (post-exacerbation) Nutritional supplementation (ONS, dietitian-led)	Pharmacological agents to prevent muscle loss: myostatin inhibitors, selective androgen receptor modulators (SARMs), activin receptor antagonists, mitochondrial/neuromuscular junction targeting agents Neuromuscular Electrical Stimulation (NMES) for individuals unable to engage in active rehabilitation
**Metabolic** **(*e.g.* hyperglycaemia, energy imbalance)**	Limit systemic corticosteroid exposure; if indicated, to 40 mg·day^−1^ for 5 days Dietetic assessment and protein- and energy-dense ONS for those at nutritional risk	Active optimisation of glycaemic control (*e.g.* insulin therapy, resistance exercise, dietary carbohydrate restriction)

ACS: acute coronary syndrome; HF: heart failure; *S*_pO_2__: oxygen saturation measured by pulse oximetry; CV: cardiovascular; ECG: electrocardiogram; BP: blood pressure; CVD: cardiovascular disease; CAD: coronary artery disease; AF: atrial fibrillation; NT-proBNP: N-terminal pro-brain natriuretic peptide; CAM: Confusion Assessment Method; MoCA: Montreal Cognitive Assessment; ONS: oral nutritional supplements.

## Summary

AECOPD disrupts multiple systems and requires timely and effective management to limit systemic damage and long-term consequences. While evidence-based individualised acute care is essential, proactive assessment of cardiopulmonary risk, limiting use of pro-catabolic treatments and post-exacerbation rehabilitation are key to recovery and reducing future risks. Emerging therapies targeting inflammatory processes and muscle loss offer promise for enhancing outcomes.

## Supplementary material

**Please note:** supplementary material is not edited by the Editorial Office, and is uploaded as it has been supplied by the author. Supplementary material 01725-2025.SUPPLEMENT
