# Supplementary material for: The systemic impact of acute exacerbations of COPD requiring hospitalisation: a narrative review
Source: ERJ Open Res. 2026 Jun 8;12(3):01725-2025. doi: 10.1183/23120541.01725-2025 (PMC13244207; doi:10.1183/23120541.01725-2025)
Supplement: Supplementary file 1 [file 01725-2025.SUPPLEMENT.pdf]

Online supplement

Search terms:

**OLS Table 1.** Search terms	
Cardiovascular	(Core set)<br>AND<br>("myocardial infarction" OR "coronary" OR "stroke" OR "cerebrovascular" OR "arrhythmia*" OR "troponin" OR "cardiovascular" OR "cardiac" OR "ischemia").mp.<br>AND<br>("incidence" OR "risk" OR "prevalence" OR "outcome*" OR "features" OR "pathophysiology" OR "mechanism*" OR "pathogenesis" OR "inflammation" OR "hypoxemia" OR "hypercapnia" OR "oxidative stress" OR "endothelial dysfunction" OR "thrombosis" OR "coagulopathy" OR "beta-blocker*" OR "statin*" OR "aspirin" OR "antiplatelet" OR "anticoagulant" OR "ACE inhibitor*" OR "angiotensin receptor blocker*" OR "calcium channel blocker*" OR "rehabilitation" OR "oxygen therapy").mp.<br>Limit to: English language, humans, yr="1995 - Current".
Cognitive	(Core set)<br>AND<br>("cognition" OR "cognitive" OR "delirium" OR "confusion" OR "disorientation" OR "memory" OR "dementia" OR "Alzheimer*" OR "mild cognitive impairment" OR "neurocognitive" OR "attention" OR "executive function").mp.<br>AND<br>("incidence" OR "risk" OR "prevalence" OR "outcome*" OR "features" OR "pathophysiology" OR "mechanism*" OR "inflammation" OR "hypoxemia" OR "hypercapnia" OR "oxidative stress" OR "endothelial dysfunction" OR "neurotransmitter*" OR "amyloid" OR "tau" OR "rehabilitation" OR "cognitive therapy" OR "antidepressant*" OR "anxiolytic*" OR "antipsychotic*" OR "cholinesterase inhibitor*" OR "pharmacolog*" OR "drug therap*").mp.<br>Limit to: English language, humans, yr="1995 - Current".
Skeletal Muscle	(Core set)<br>AND<br>("skeletal muscle" OR "quadriceps" OR "sarcopenia" OR "cachexia" OR "myopathy" OR

	"atrophy" OR "weakness" OR "fatigue" OR "handgrip" OR "grip strength" OR "sit-to-stand" OR "physical performance" OR "mobility" OR "exercise capacity").mp.<br>AND<br>("incidence" OR "risk" OR "prevalence" OR "outcome*" OR "features" OR "pathophysiology" OR "mechanism*" OR "atrophy" OR "mitochondria*" OR "oxidative stress" OR "inflammation" OR "anabolic resistance" OR "protein synthesis" OR "nutritional supplement*" OR "protein" OR "amino acid*" OR "anabolic" OR "selective androgen receptor modulator*" OR "SARM*" OR "testosterone" OR "steroid*" OR "creatine" OR "physiotherapy" OR "resistance training" OR "rehabilitation" OR "exercise therap*" OR "pharmacolog*" OR "drug therap*").mp.<br>Limit to: English language, humans, yr="1995 - Current".
Metabolic	(Core set)<br>AND<br>("metabolic syndrome" OR "insulin resistance" OR "diabetes" OR "type 2 diabetes" OR "glucose intolerance" OR "hyperglycemia" OR "hyperlipidemia" OR "dyslipidemia" OR "obesity" OR "BMI" OR "body mass index" OR "adiposity" OR "metabolism").mp.<br>AND<br>("incidence" OR "risk" OR "prevalence" OR "outcome*" OR "features" OR "pathophysiology" OR "mechanism*" OR "inflammation" OR "oxidative stress" OR "insulin sensitivity" OR "glucose uptake" OR "lipid metabolism" OR "mitochondria*" OR "nutritional intervention*" OR "diet" OR "exercise therap*" OR "lifestyle intervention*" OR "rehabilitation" OR "weight loss" OR "antidiabetic" OR "metformin" OR "glucose lowering" OR "statin*" OR "drug therap*" OR "pharmacolog*").mp.<br>Limit to: English language, humans, yr="1995 - Current".
